# Water‐Soluble Iron Porphyrins as Catalysts for Suppressing Chlorinated Disinfection Byproducts in Hypochlorite‐Dependent Water Remediation

**DOI:** 10.1002/cssc.202402171

**Published:** 2025-01-10

**Authors:** Silène Engbers, Maja J. Lind, Mathias L. Skavenborg, Johannes E. M. N. Klein, Frants R. Lauritsen, Christine J. McKenzie

**Affiliations:** ^1^ Molecular Inorganic Chemistry Stratingh Institute for Chemistry Faculty of Science and Engineering University of Groningen Nijenborgh 3 9747 AG Groningen The Netherlands; ^2^ Department of Physics Chemistry and Pharmacy University of Southern Denmark Campusvej 55 Odense M 5320 Denmark

**Keywords:** Porphyrins, Water remediation, Oxidation, Catalysis, Iron

## Abstract

We are facing a world‐wide shortage of clean drinking water which will only be further exacerbated by climate change. The development of reliable and affordable methods for water remediation is thus of utmost importance. Chlorine (which forms active hypochlorites in solution) is the most commonly used disinfectant due to its reliability and low cost. One drawback is that it reacts with organic pollutants to generate toxic chlorinated byproducts. To mitigate chlorination in water remediation, we have investigated the use of catalytic amounts of charged water‐soluble iron porphyrins. These are known to activate hypochlorite to generate high valent oxoiron species. We studied the depletion of the model micropollutant phenol and the accumulation of chlorinated disinfection byproducts under water remediation conditions, using iron porphyrins [(TMPyP)FeCl]Cl_4_ and (NH_4_)_4_[(TPPS)FeCl] as catalysts, by membrane inlet mass spectrometry. Despite bearing opposite charges on the meso‐substituent, both iron porphyrins suppress the formation of chlorinated disinfection by‐products equally well. To gain further insight, spectroscopic studies were performed. These showed the transient formation of Compound II, followed by either regeneration of the iron(III) porphyrin at low NaOCl concentrations, or total decomposition of the porphyrin complex at high NaOCl concentrations. Potential future directions for modifications of porphyrin‐based catalysts are discussed.

## Graphical abstract

Water remediation using chlorine (for which hypochlorite is the active disinfectant) has the disadvantage of degrading micropollutants to chlorinated compounds. To bypass this and promote total mineralization of the model micropollutant phenol to CO_2_ and H_2_O, we investigated the use of catalytic amounts of iron porphyrins in hypochlorite‐dependent water remediation. Porphyrins bearing positive and negative charges on the *meso*‐substituent are both active for the suppression of accumulation of chlorinated byproducts, without hindering the degradation of phenol.

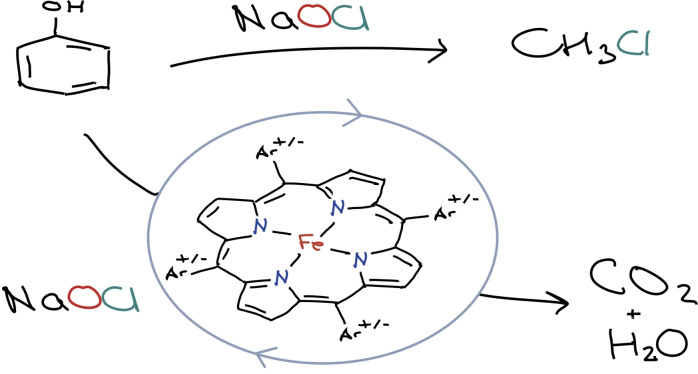

## Introduction

Safe drinking water is a basic human need. We are currently facing a critical world‐wide shortage of clean water that is being further escalated by climate change.^[1]^ In 2020, a quarter of the global population lacked safely managed drinking water services.^[2]^ Notably this is not limited to developing countries. Even in the United States, unsafe drinking water leads to about 7 million waterborne illnesses and over 6600 deaths annually.^[3]^ To indicate the urgency of this matter, the United Nations has made their 6^th^ sustainable development goal of the 2030 Agenda for Sustainable Development to “ensure availability and sustainable management of water and sanitation for all”.^[4]^

To overcome the challenge of providing safe drinking water on a global scale, we need reliable and affordable methods for water remediation. Common techniques for disinfection rely on the use of chlorine, monochloramine, chlorine dioxide, or ozone.^[5]^ Chlorine is the most commonly used disinfectant in both developing and developed countries due to its versatility, convenience, and economic viability.[^5^, ^6^] Chlorine gas (Cl_2_) will react with water to form hypochlorites (^−^OCl), and at pH values above 3 extremely little Cl_2_ remains in solution.^[6]^ Hypochlorite is thus the active disinfecting agent when chlorine is applied in water remediation.

**Scheme 1:**
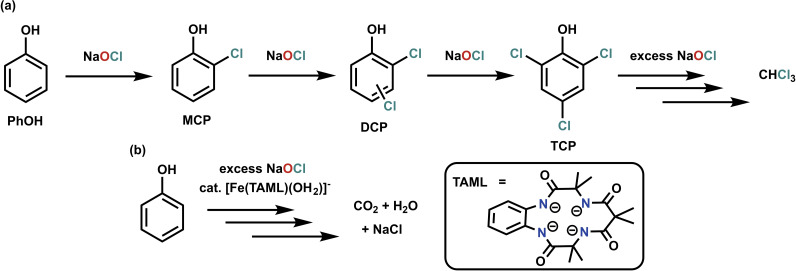
Proposed pathways for the hypochlorite‐mediated total mineralization of PhOH in the absence (**a**) and presence (**b**)^[14]^ of catalytic amounts of [Fe(TAML)(OH_2_)]^−^.

One unfortunate consequence of disinfecting water with chlorine is its reaction with naturally occurring organic molecules and micropollutants to form (toxic and carcinogenic)^[7]^ halogenated organic compounds, (e. g. trihalomethanes and chlorophenols).^[5]^ Many studies have focused on understanding and quantifying the accumulation of disinfection byproducts (DBPs).[^8^, ^9^, ^10^, ^11^, ^12^] One convenient method is the use of membrane inlet mass spectrometry (MIMS).^[12]^ Hereby the accumulation of DBPs can be monitored directly over time from the reaction mixture at relevant concentrations and in tap water. A recent study from one of our groups used MIMS to monitor the reaction of NaOCl with phenol (PhOH) as a model micropollutant, identifying monochlorophenol (MCP), dichlorophenol (DCP), trichlorophenol (TCP), chloroform (CHCl_3_), and eventually bromo‐dichloromethane (BDM) as the main DBPs that accumulate in solution (Scheme 1a).^[13]^ The unexpected bromination product originates from naturally occurring bromide in tap water.^[13]^

Several studies on the degradation of model micropollutants have employed iron TAML (TAML=a tetra‐amido macrocyclic ligand, see Scheme 1b) compounds as active catalysts together with hydrogen peroxide (H_2_O_2_) as the oxidant.[^15^, ^16^, ^17^, ^18^, ^19^] Replacing H_2_O_2_ by NaOCl was recently shown to significantly enhance the reaction rate.^[20]^ With this in mind, one of our groups employed catalytic amounts of [Fe(TAML)(OH_2_)]^−^ in the hypochlorite‐dependent water remediation reaction with PhOH as a model micropollutant. It was found that [Fe(TAML)(OH_2_)]^−^ significantly suppresses the accumulation of chlorinated DBPs without hindering the decomposition of PhOH.^[14]^ [Fe(TAML)(OH_2_)]^−^ reacts with NaOCl to form an oxoiron(V) species,^[21]^ which is a potent oxidant (e. g. for hydrogen atom abstraction and oxygen atom transfer reactions).[^22^, ^23^, ^24^, ^25^, ^26^, ^27^, ^28^] It is thus postulated that [Fe(TAML)(OH_2_)]^−^ activates hypochlorite to allow total mineralization of PhOH to CO_2_ and H_2_O instead of CHCl_3_ (Scheme 1b).^[14]^

The TAML complexes were originally designed to replicate the reactivity of peroxidases, a class of enzymes that uses hydrogen peroxide as a substrate.[^22^, ^29^] Chloroperoxidase, which similarly to peroxidase is a heme‐containing enzyme, transiently generates hypochlorites. By doing so, it can achieve the two electron oxidation of chloride and thereby perform electrophilic chlorinations.[^30^, ^31^] An important step in the catalytic pathway of chloroperoxidase is the attack of chloride onto the oxido ligand of Compound I (the general term for an oxoiron(IV) with a one‐electron oxidized ring),^[32]^ thereby forming an iron(III) hypochlorite.^[30]^

**Scheme 2:**
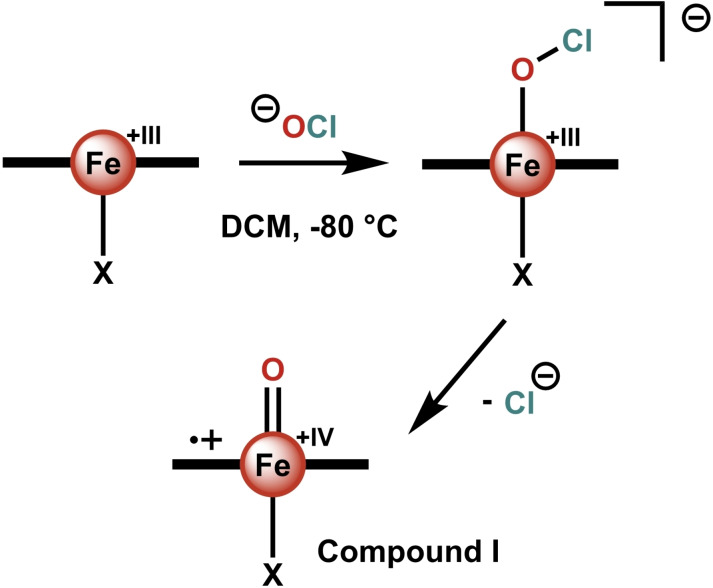
The reported reactivity of *meso*‐substituted porphyrins with hypochlorite in organic solvents.^[35]^

In organic solvents, aryl‐*meso*‐substituted iron(III) porphyrins, which can be considered bioinspired^[33]^ structures of chloroperoxidase, will react with hypochlorite at low temperatures to form a short lived iron(III) hypochlorite species (Scheme 2).[^34^, ^35^] This will rapidly decompose to Compound I,^[35]^ a pathway that can be considered as the microscopic reverse of the formation of an iron(III) hypochlorite in chloroperoxidase. Compound I is a potent oxidant^[36]^ containing the same oxidative equivalents as the TAML oxoiron(V). It was thus envisioned that iron porphyrins may equally be viable catalysts to avoid the accumulation of chlorinated DBPs in hypochlorite‐mediated water remediation. Furthermore, Compound I has been shown to oxidatively dechlorinate trichlorophenol in enzymatic[^37^, ^38^, ^39^, ^40^, ^41^, ^42^, ^43^] and molecular[^44^, ^45^, ^46^, ^47^, ^48^, ^49^, ^50^, ^51^] systems, providing a method to decompose chlorinated disinfection byproducts.

One benefit of simple *meso*‐substituted porphyrins is their straightforward electronic tunability through variation of the *meso*‐substituent. Even relatively small variations of the *meso*‐substituent have been shown to significantly influence the reactivity of iron porphyrins.[^35^, ^52^, ^53^] For reactions in aqueous media, porphyrins with charged *meso*‐substituents are usually employed, leading to a choice of positive or negative charge. Positive charge on the meso‐substituent has been correlated with increased redox potentials and the resulting reactivity of Compound I.^[54]^ Contrasting this, introduction of negative charge on the *meso*‐substituent has been suggested to increase the stability and lifetime of the typically short‐lived Compound I.^[55]^ Considering that water remediation is performed at very high dilutions, we envisioned that there may be a subtle interplay between the necessity of a high activity and high stability of Compound I for this particular application.

## Results and Discussion

**Figure 1:**
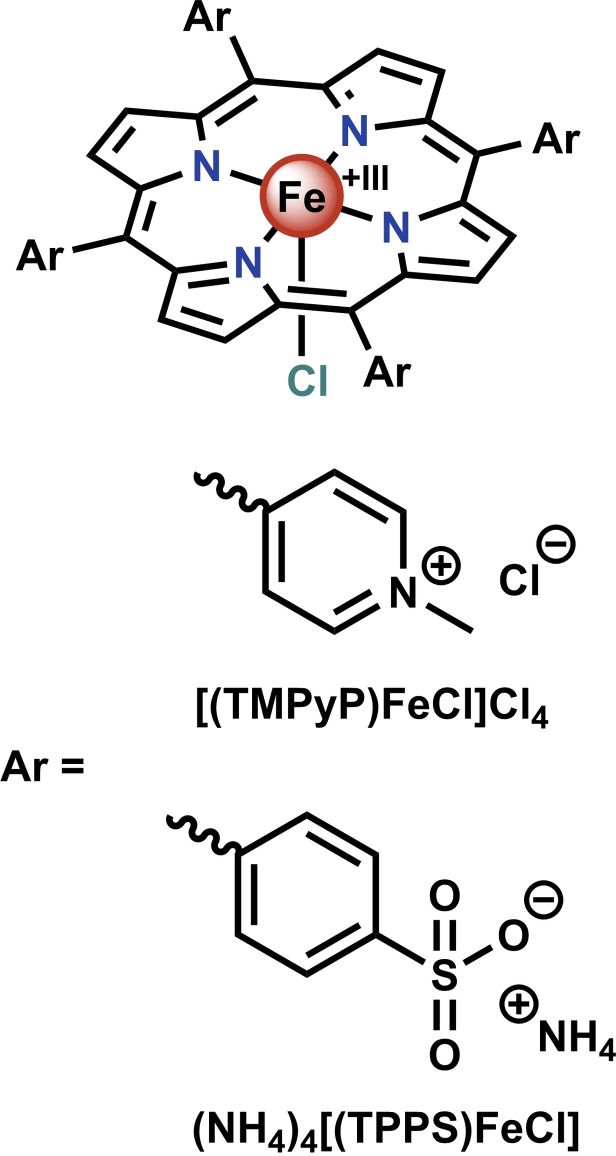
Structures of the porphyrins studied in this work: [(TMPyP)FeCl]Cl_4_ and (NH_4_)_4_[(TPPS)FeCl].

To probe the viability of iron porphyrins as catalysts to suppress chlorination in hypochlorite‐dependent water remediation, we used MIMS to follow the reaction of PhOH with 7 eq. NaOCl in the presence of catalytic amounts of positively charged [(TMPyP)FeCl]Cl_4_ (where TMPyP=5,10,15,20‐tetra(4‐*N*‐methylpyridyl)porphyrin) or negatively charged (NH_4_)_4_[(TPPS)FeCl] (where TPPS=5,10,15,20‐tetra(4‐sulphonatophenyl)porphyrin) in tap water^[56]^ (see Figure 1 for their structures). Spectroscopic analyses were further performed to inspect both the intermediates formed and species that accumulate upon addition of NaOCl to both iron porphyrins in tap water, as well as overall stability of the iron porphyrins.

Although previous analysis of the reaction of PhOH with NaOCl in tap water by MIMS had identified halogenated phenols (MCP, DCP, and TCP), as well as other small halogenated molecules (CHCl_3_ and BDM) as main DBPs,[^13^, ^14^] we decided to only follow MCP, DCP and TCP in the current study. These three products are the first direct products in the reaction of NaOCl with PhOH and form rapidly. We therefore envisioned that these would give us the most direct information on the activity of the [(TMPyP)FeCl]Cl_4_ and (NH_4_)_4_[(TPPS)FeCl] porphyrin complexes.

**Figure 2:**
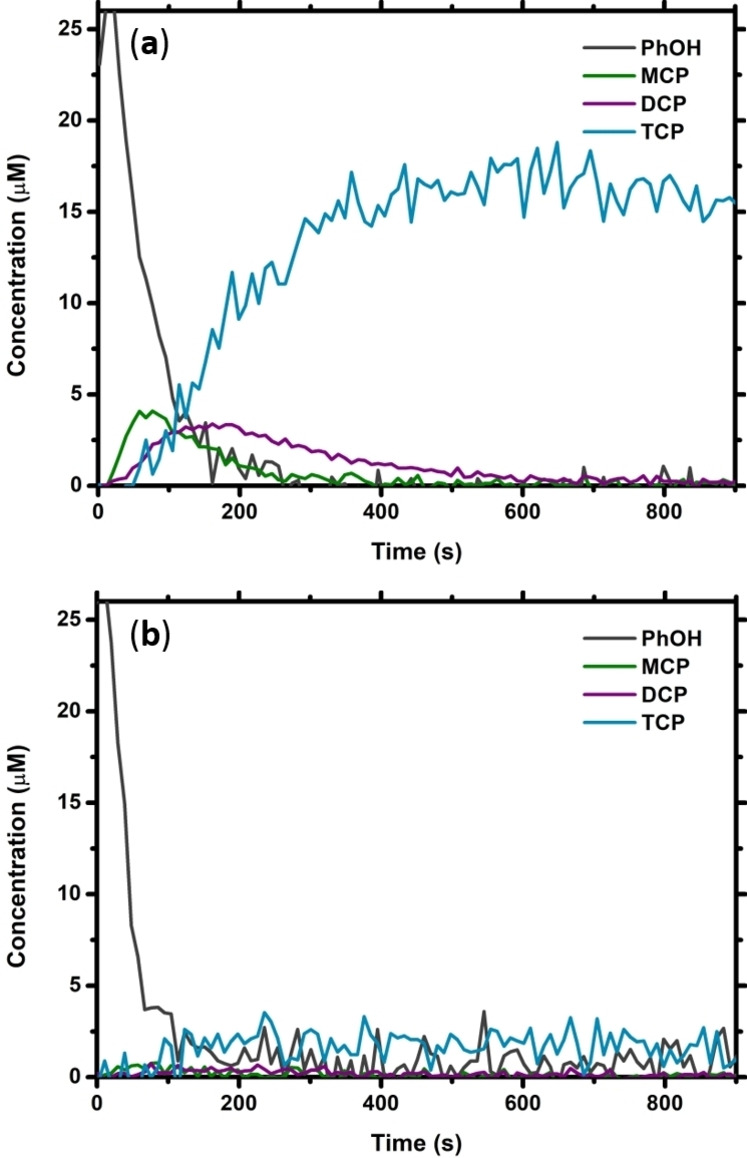
Time traces of the quantified MIMS data showing the depletion of PhOH (dark grey trace, initially 25 μM) after addition of 7 eq. NaOCl at t=0 s, and the accumulation of MCP, DCP and TCP (green, purple, and blue traces, respectively) (**a**) in the absence of catalyst, and (**b**) in the presence of 4 mol % (NH_4_)_4_[(TPPS)FeCl]. Both reactions were performed in tap water at 40 °C.

Figure 2a shows a typical time trace of the depletion of PhOH (25 μM) by 7 eq. NaOCl in tap water in the absence of catalyst at 40 °C and the resulting accumulation of MCP, DCP, and TCP. As would be expected (see Scheme 1a), MCP accumulates fastest, followed by DCP and TCP. Although both MCP and DCP are fully depleted within 600 s, the concentration of TCP has not reached its theoretical maximum concentration of 25 μM at this point, and already starts slowly depleting. This is due to the reaction of TCP into fragments which are not identifiable by MIMS,^[13]^ and then further to CHCl_3_, which has not been followed in this experiment. BDM only starts accumulating (in small quantities) after 20 minutes of reaction time,^[13]^ and so would not be detectable within the time‐frame of these experiments.

**Figure 3:**
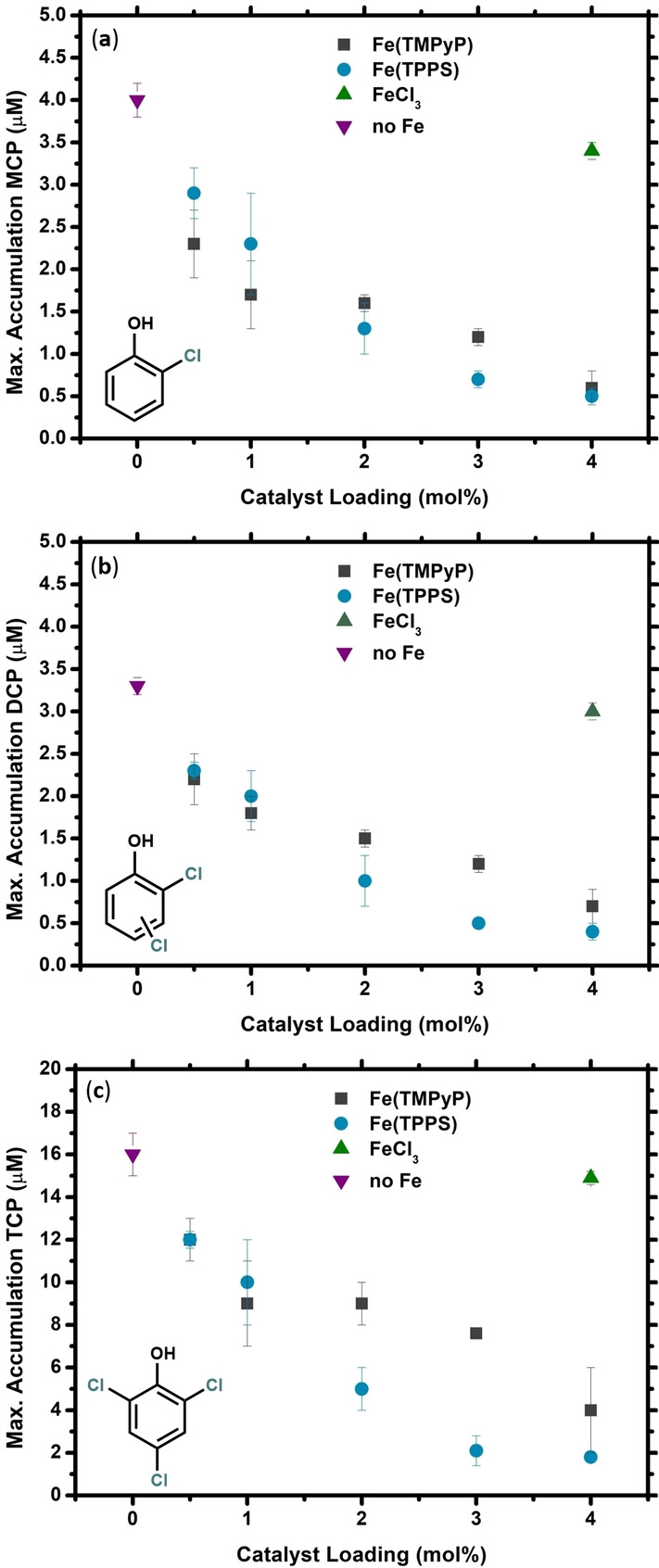
Maximum accumulation of MCP (**a**), DCP (**b**), and TCP (**c**) as a function of catalyst loading. Data points for [(TMPyP)FeCl]Cl_4_ are indicated by dark grey squares, those for (NH_4_)_4_[(TPPS)FeCl] are indicated by blue circles, the data points for FeCl_3_ are indicated by a green triangle, and the data for the reference (uncatalyzed reaction) is indicated by a purple upside‐down triangle. Reactions were performed in tap water at 40 °C with an initial PhOH concentration of 25 μM NaOCl (7 eq.) was added to initiate the reaction and its progress was followed by MIMS. All data points were performed in triplicate. Exact values can be found in Table S5.

The addition of 4 mol % (NH_4_)_4_[(TPPS)FeCl] to the reaction mixture results in almost no MCP or DCP accumulation, and only little accumulation of TCP (only achieving a maximum concentration of around 2 μM), see Figure 2b. Similar behavior is observed for the addition of 4 mol % [(TMPyP)FeCl]Cl_4_ (Figure S1a), although the maximum accumulation of TCP is found to be slightly higher (around 5 μM). The catalyst loading was lowered to 3, 2, 1, and 0.5 mol % to assess how strongly the DBP suppression depends on the amount of iron porphyrin added. The resulting maximum accumulations of MCP, DCP, and TCP after a triplicate run of each reaction can be found in Figure 3 (and Table S5), alongside the results for the uncatalyzed reaction as well as a control reaction with FeCl_3_.

It is clear that increasing catalyst loading decreases the maximum accumulation of MCP, DCP, and TCP, for both [(TMPyP)FeCl]Cl_4_ and (NH_4_)_4_[(TPPS)FeCl]. Overall, there does not seem to be a significant difference in activity between [(TMPyP)FeCl]Cl_4_ and (NH_4_)_4_[(TPPS)FeCl] for this reaction. Only at catalyst loadings of 2–3 mol % does (NH_4_)_4_[(TPPS)FeCl] slightly outperform [(TMPyP)FeCl]Cl_4_, particularly for the suppression TCP. For any practical water remediation application, in which the exact concentration of micropollutant is generally unknown or even variable over time, there is no significant benefit in the use of one iron porphyrin over the other. One possible explanation for this is that the enhanced reactivity of Compound I derived from [(TMPyP)FeCl]Cl_4_ is counteracted by its short life‐time.

As a control experiment to verify that the iron porphyrins are performing the reaction and not an iron salt generated from their decomposition, the reaction was similarly performed in the presence of 4 mol % FeCl_3_ (Figure 3, Table S5, and Figure S1b). No significant suppression of chlorinated byproduct was observed in this case, suggesting that the iron porphyrins are indeed the active catalyst and that a simple iron salt is not a suitable substitute.[^57^, ^58^]

Iron porphyrins are known to readily form μ‐oxo bridged diiron complexes (typically referred to as μ‐oxo dimers in the literature) under certain conditions.[^59^, ^60^, ^61^] Moreover, non‐heme μ‐oxo bridged diiron compounds have been shown to react with hypochlorite.^62^ With this in mind, it was envisioned that a mixture of positively charged and negatively charged iron porphyrins may enhance or reduce the observed reactivity. A 1 : 1 mixture of [(TMPyP)FeCl]Cl_4_ and (NH_4_)_4_[(TPPS)FeCl], where the total iron porphyrin concentration equated 2 or 3 mol % catalyst loading, was thus employed in the reaction (Figure S2 and Table S6). The maximum concentrations of DBPs were all approximately within the range expected if only one of the two iron porphyrins had been used. Mixing these two iron porphyrins thus does not significantly affect the suppression of chlorination. This may suggest that μ‐oxo bridged compounds are not formed in this case.

It is worth mentioning that although both iron porphyrins are active in the suppression of chlorinated byproducts, they do not perform as efficiently as the [Fe(TAML)(OH_2_)]^−^ previously reported.^[14]^ The iron porphyrins still show some accumulation of chlorinated phenols at 4 mol % catalyst loading when spiking tap water with 25 μM PhOH. The [Fe(TAML)(OH_2_)]^−^ suppresses chlorination to below the detection limit of chlorophenols at only 0.2 mol % catalyst loading when spiking tap water with 16 μM PhOH.^[14]^

The TAML ligand bears some similarities with porphyrins in that they are both planar cyclic ligands coordinating to a metal through four nitrogen donor atoms. However, the TAML is a stronger σ‐donor than the porphyrin, tetra‐anionic rather than di‐anionic, and less redox active. As a result, assuming that in both cases heterolytic FeO‐Cl bond cleavage is achieved, the oxidative equivalents are constrained to the metal in the case of the TAML ligand (generating an Fe(V) oxo species) instead of shared between the metal and the ligand (Compound I, see Scheme 2). This difference in active species may be the cause of this substantial contrast in activity. However, in the presence of such a large excess of NaOCl (1400 eq. at 0.5 mol % catalyst loading), it is wise to consider catalyst stability.

**Figure 4:**
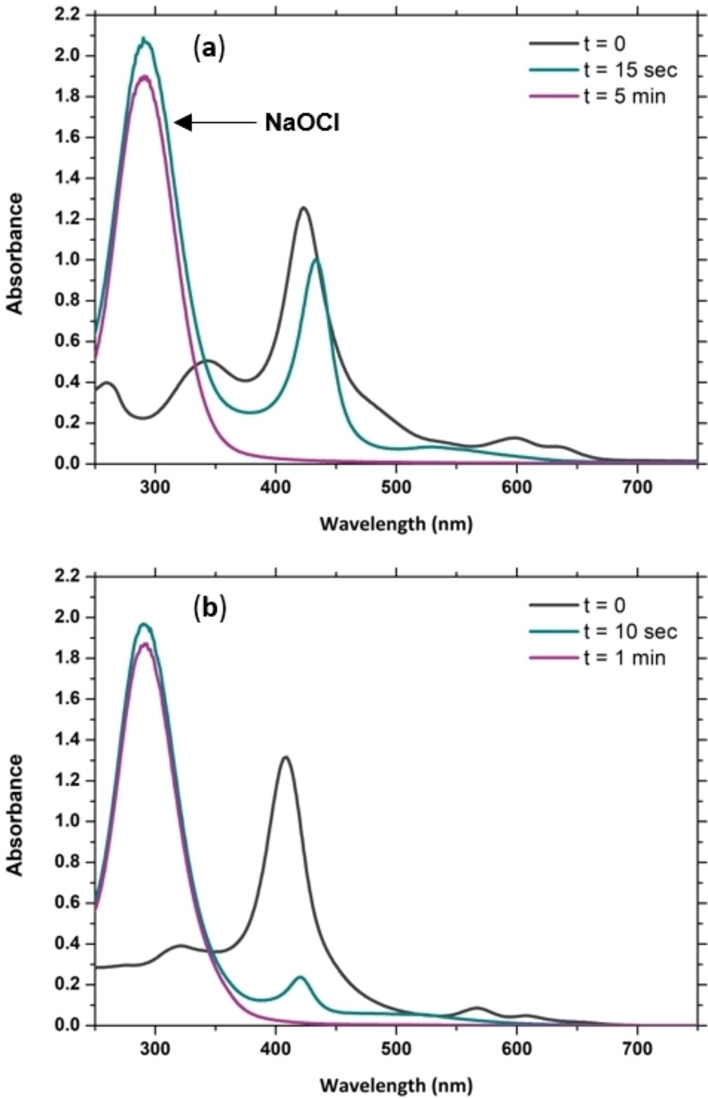
UV‐Vis absorption spectra of the reactions of (a) [(TMPyP)FeCl]Cl_4_ and (b) (NH_4_)_4_[(TPPS)FeCl] (both 20 μM) with 334 eq. NaOCl in tap water at 20 °C. The band at 291 nm corresponds to NaOCl.

To investigate the stability of [(TMPyP)FeCl]Cl_4_ and (NH_4_)_4_[(TPPS)FeCl] in the presence of excess NaOCl, UV‐Vis experiments were performed wherein 334 eq. NaOCl was added to 20 μM of iron porphyrins in tap water at 20 °C (Figure 4). It should foremost be noted that the UV‐Vis spectra observed upon dilution of both [(TMPyP)FeCl]Cl_4_ and (NH_4_)_4_[(TPPS)FeCl] to 20 μM in tap water do not correspond to those expected for porphyrins bearing a chloride as axial ligand. Porphyrins bearing chloride as an axial ligand are known to have two dominant Q‐bands at approximately 510 and 640 nm.[^52^, ^63^] When dissolving the iron porphyrins in deionized water instead of tap water, we could identify such spectra (Figure S3). In the case of [(TMPyP)FeCl]Cl_4_, diluting from 200 μM to 5 μM in deionized water showed a gradual change of the spectrum with Q bands at 513 and 634 nm to that found in tap water, indicating a direct relationship between the two species. For (NH_4_)_4_[(TPPS)FeCl], the spectrum with Q bands at 528 nm and 654 nm persists even at 5 μM in deionized water. The spectra that we observe in tap water are consistent with those found upon dilution of [(TMPyP)FeCl]Cl_4_ and (NH_4_)_4_[(TPPS)FeCl] in buffered water.[^60^, ^61^, ^64^, ^65^] These spectra have been reported to arise from an equilibrium between the corresponding (porphyrin)Fe(OH_2_)_2_ and (porphyrin)Fe(OH_2_)(OH), and further involving the corresponding μ‐oxo bridged diiron compounds at high pH values.[^60^, ^61^, ^64^, ^65^]

For both iron porphyrins, upon addition of NaOCl, transient formation of an intermediate was observed before complete decomposition of the porphyrin (Figure 4). This process is 5 times faster for (NH_4_)_4_[(TPPS)FeCl] than for [(TMPyP)FeCl]Cl_4_ (complete within 1 instead of 5 minutes). It should be noted that the concentrations required for UV‐Vis analysis are at least one order of magnitude higher than those used for the water remediation experiments monitored by MIMS (in which the iron porphyrin concentrations range from 125 nm to 1 μM). We could thus expect that the decomposition rate observed by UV‐Vis is much higher than in the MIMS experiments.

**Figure 5:**
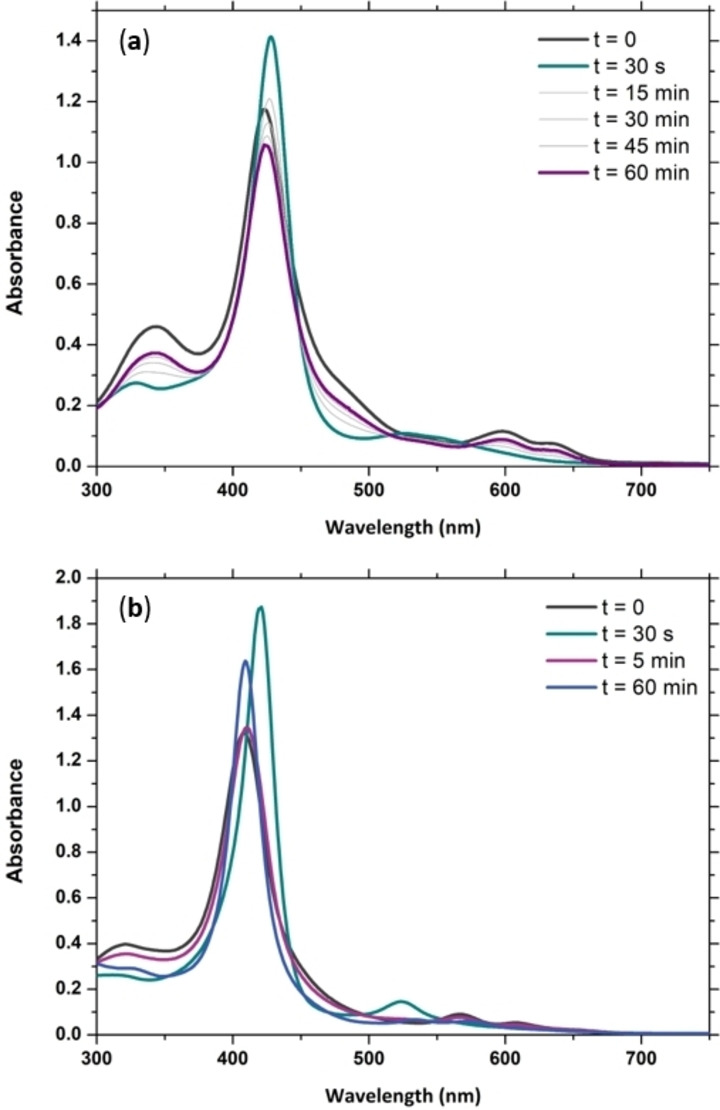
UV‐Vis absorption spectra of the reactions of (**a**) [(TMPyP)FeCl]Cl_4_ and (**b**) (NH_4_)_4_[(TPPS)FeCl] (both 20 μM) with 2 eq. NaOCl in tap water at 20 °C.

To slow down the reaction and more closely observe the intermediate formed during the reaction, the amount of NaOCl was decreased to 2 equivalents (Figure 5). For both [(TMPyP)FeCl]Cl_4_ and (NH_4_)_4_[(TPPS)FeCl] rapid formation of the transient species is observed upon addition of NaOCl. These transient species show UV‐Vis spectra consistent with those previously reported for respective the oxoiron(IV) species^[32]^ (Compound II).^[61]^ Compound II of TMPyP slowly decomposes back to its parent compound over 60 minutes, with a small loss of absorption likely due to NaOCl mediated decomposition. Compound II of TPPS rapidly decomposes back to its parent compound within 5 minutes, and subsequently further transforms to a new species over the next 55 minutes. Although the identity of this new species is unclear, addition of further 2 equivalents of NaOCl does not lead to any accumulation of Compound II (Figure S4).

The formation of Compound II can arise from two separate pathways. Either the FeO‐Cl bond is cleaved homolytically to directly generate Compound II, or the FeO‐Cl bond is cleaved heterolytically to initially form Compound I which is rapidly decomposed to Compound II. This second option has been observed in organic solvents for porphyrins bearing strongly electron‐withdrawing *meso*‐substituents,^[35]^ and in aqueous solutions for [(TMPyP)FeCl]Cl_4_ when *meta*‐chloroperbenzoic acid (mCPBA) is employed as oxidant.^[66]^

In attempts to observe transient formation of Compound I, the reaction of the iron porphyrins with 2 equivalents of NaOCl at 7 °C in tap water was followed by stopped flow (Figure S5). No accumulation of Compound I could be observed for either [(TMPyP)FeCl]Cl_4_ and (NH_4_)_4_[(TPPS)FeCl]. It is also interesting to note that although the degradation is faster for (NH_4_)_4_[(TPPS)FeCl] than [(TMPyP)FeCl]Cl_4_ (Figure 4), the rate of formation of Compound II is much slower for (NH_4_)_4_[(TPPS)FeCl] (Figure S5). Considering that it is well known that Compound I is short‐lived and difficult to observe, especially in aqueous media where the temperature cannot be lowered as much as in organic solvents,^[55]^ it remains inconclusive whether homolytic or heterolytic FeO‐Cl bond cleavage occurs in this case.

## Conclusions and Implications

In conclusion, [(TMPyP)FeCl]Cl_4_ and (NH_4_)_4_[(TPPS)FeCl] were found to be active in the suppression of the chlorination of PhOH in hypochlorite‐dependent water remediation experiments. Surprisingly, despite the difference in charge on the *meso*‐substituent, the two iron porphyrins perform comparably. Increasing the catalyst loading systematically decreases the maximum accumulation chlorinated byproducts. When 4 mol % catalyst loading is added to a water remediation experiment spiked with 25 μM PhOH, both [(TMPyP)FeCl]Cl_4_ and (NH_4_)_4_[(TPPS)FeCl] suppress the maximum accumulation of MCP and DCP to below 1 μM, and the maximum accumulation of TCP to below 5 μM. For practical applications, the porphyrin concentration needs to be sub‐nanomolar to avoid for example phototoxic effects to the body.^[67]^ Using a 1 : 1 mixture of [(TMPyP)FeCl]Cl_4_ and (NH_4_)_4_[(TPPS)FeCl] does not appear to improve nor diminish performance.

Spectroscopic investigations show that upon addition of excess (334 eq.) NaOCl both iron porphyrins rapidly degrade to leave a featureless UV‐Vis spectrum. In contrast to the results from the water remediation experiments, we observe a difference in reactivity between the two iron porphyrins, with (NH_4_)_4_[(TPPS)FeCl] degrading 5 times faster than [(TMPyP)FeCl]Cl_4_. Thus there must be several counterbalancing factors, leading to the similar reactivities observed in the water remediation experiments. Reducing the equivalents of NaOCl to 2 allows the identification of Compound II as the main intermediate formed. Under these conditions, the iron porphyrins do not fully decompose. Compound II is mainly transformed back into parent iron porphyrin species, with minor loss to porphyrin degradation. It is important to note that spectroscopic studies need to be performed at concentrations at least one order of magnitude higher than those employed for the water remediation experiments. It is thus difficult to quantify the rate of porphyrin degradation in the water remediation experiments. Although low stability is likely a factor that will limit the activity of the iron porphyrins, the fact that they do degrade is beneficial for water remediation purposes. An important feature of a water remediation catalyst is that they do not persist in solution after their intended function, such as to avoid their consumption.^[68]^

Compound I holds more oxidative equivalents than Compound II, making it a more potent oxidant and thus a more suitable active species for total mineralization of PhOH to CO_2_ and H_2_O. Based on previous literature reports, we had envisioned the formation of Compound I to be feasible under the conditions employed.[^35^, ^55^, ^66^] Despite our best efforts, we were unable to observe any accumulation of Compound I. This does not in itself mean that Compound I is not formed, and the lack of detection could instead be due to its known low stability and short lifetime. On the other hand, it is not impossible that the FeO‐Cl bond cleaves homolytically, leading to the direct formation of Compound II. Further exploration and optimization of iron porphyrin catalysts would thus benefit from addressing the accessibility and stability of Compound I as well as increased stability of the porphyrin to oxidative conditions.

## Conflict of Interests

The authors declare no conflict of interest.

1

## Supporting information

As a service to our authors and readers, this journal provides supporting information supplied by the authors. Such materials are peer reviewed and may be re‐organized for online delivery, but are not copy‐edited or typeset. Technical support issues arising from supporting information (other than missing files) should be addressed to the authors.

Supporting Information

## Data Availability

The data that support the findings of this study are openly available in dataverse.nl at https://doi.org/10.34894/IWS45X, reference number 69.
