# Supplementary material for: Water‐Soluble Iron Porphyrins as Catalysts for Suppressing Chlorinated Disinfection Byproducts in Hypochlorite‐Dependent Water Remediation
Source: ChemSusChem. 2025 Jan 10;18(9):e202402171. doi: 10.1002/cssc.202402171 (PMC12051255; doi:10.1002/cssc.202402171)
Supplement: Supplementary file 1 — Supporting Information [file CSSC-18-e202402171-s001.pdf]

ChemSusChem

Supporting Information

Water-Soluble Iron Porphyrins as Catalysts for Suppressing Chlorinated Disinfection Byproducts in Hypochlorite-Dependent Water Remediation

Silène Engbers, Maja J. Lind, Mathias L. Skavenborg, Johannes E. M. N. Klein,*
Frants R. Lauritsen,* and Christine J. McKenzie*

Supporting Information

for

Water-Soluble Iron Porphyrins as Catalysts for Suppressing Chlorinated Disinfection Byproducts in Hypochlorite-Dependent Water Remediation

Silène Engbers,[a] Maja J. Lind,[b] Mathias L. Skavenborg,[b] Johannes E. M. N. Klein*,[a] Frants R. Lauritsen,*,[b] and Christine J. McKenzie*,[b]

[a] Molecular Inorganic Chemistry, Stratingh Institute for Chemistry, Faculty of Science and Engineering, University of Groningen, Nijenborgh 3, 9747 AG Groningen (The Netherlands). E-mail: j.e.m.n.klein@rug.nl

[b] Department of Physics, Chemistry and Pharmacy, University of Southern Denmark, Campusvej 55, 5320 Odense M (Denmark). Email: frl@sdu.dk; mckenzie@sdu.dk

Table of Contents

**1. General Considerations**	**S2**
**2. Methodology for DBP Quantification using MIMS**	**S2**
**3. General Procedure for MIMS Experiments**	**S3**
**4. Additional MIMS Data**	**S5**
**5. General Procedures for UV-Vis and Stopped Flow Experiments**	**S7**
**6. Additional Spectroscopic Data**	**S7**
**7. Data Availability**	**S8**
**8. References**	**S9**

1. General Considerations

Unless stated otherwise, all chemicals were obtained commercially and used without further purification. Unless stated otherwise all reactions were performed in tap water obtained directly from the tap at the University of Southern Denmark, Odense. Drinking water in Odense, Denmark, does not contain residual chlorine or halogenated disinfection byproducts, it is supplied from groundwater reservoirs and disinfected by ultraviolet irradiation. 5,10,15,20-(tetra-N-methyl-4-pyridyl)porphyrin iron(III) penta-chloride, [(TMPyP)FeCl)]Cl_4, and 5,10,15,20-(tetra-4-sulfonatophenyl)porphyrin iron(III) chloride tetra-ammonium, $(NH_4)_4$[(TPPS)FeCl)], were purchased from PorphyChem. Sodium hypochlorite (5% active chlorine, which is calculated to equal 764 mM) was purchased from Thermo Scientific. Phenol (99%) was purchased from Supelco, 4-Chlorophenol (MCP)and 2,4-dichlorophenol (DCP) where purchased from Merck as 100 mg pure certified standards, and 2,4,6-Trichlorophenol (TCP) 99% was purchased from Fluka. $FeCl_3$ (>98%) from Bie & Berntsen.

Membrane inlet mass spectrometry (MIMS) data were collected using a Prisma Pro quadrupole mass spectrometer (Pfeiffer Vacuum, Germany), with a silicone membrane (0.127 mm, Sil-Tec sheeting, Technical Products, USA) separating the solution in the reaction chamber from the mass spectrometer vacuum chamber.

UV-Vis spectra were recorded on an Agilent HP8453 UV-Vis spectrophotometer equipped with a temperature control from Quantum Northwest. Stopped flow experiments were performed using the Applied Photophysics SX20 Stopped Flow Spectrometer equipped with a photodiode array accessory.

2. Methodology for DBP Quantification using MIMS

The methodology for the quantification of PhOH, MCP, DCP, and TCP by MIMS was adapted from a study previously reported.[1]

MIMS experiments were performed in selected ion mode (SIM), monitoring m/z: 94, 128, 162, and 196. These are the molecular ions and also the main m/z for PhOH, MCP, DCP, and TCP, respectively. To acquire an optimal signal to noise ration a dwell time of 1s was used for all ions except for TCP where the dwell time was 2s.

To aid quantification, the MIMS signal of tap water was measured over 5 minutes before every reaction. After this, PhOH was added (resulting in a PhOH concentration of 25 µM, see section 3) and the solution measured over 5 minutes.

The quantification of MIMS data was performed using several steps:

1) All data was background corrected by subtracting the average intensity of the first 5 minutes for the relevant m/z.
2) The sensitivity of PhOH (A μM^{-1}) was calculated by dividing the signal increase of m/z 94 by 25.
3) Intensities were corrected for overlap due to fragmentation (see Table S1).
4) Overlap corrected intensities of m/z 94 were converted to concentration of PhOH (µM) by dividing by the sensitivity of PhOH.
5) Overlap corrected intensities of m/z 128, 162, and 196, were converted to concentration of MCP, DCP, and TCP (µM), respectively by first dividing by the

appropriate sensitivity correction factor (see Table 2) to convert the recorded signal to a phenol equivalent signal. Then the phenol equivalent signals were divided by the sensitivity of PhOH.

**Table S1.** Formulas used to correct signal intensities for overlap, see previous work for explanation.[1]		
**Compound**	**m/z**	**Overlap Correction Formula**
PhOH	94	$I_{94,corr} = I_{94} – (0.09 \times I_{196})$
MCP	128	$I_{128,corr} = I_{128} – (0.05 \times I_{162})$
DCP	162	$I_{162,corr} = I_{162} – (0.09 \times I_{196})$
TCP	196	$I_{196,corr} = I_{196}$

To determine a correction factor for the sensitivity of MCP, DCP, and TCP relative to PhOH, separate solutions of known concentration (25 µM in each case) of PhOH, MCP, DCP, and TCP were analysed by MIMS over 5 minutes. The average signal intensity for the relevant m/z was noted, and the background signal of tap water directly before the measurement was subtracted from it. The signal intensity relative to that of PhOH is utilized as the correction factor for MCP, DCP, and TCP (see Table S2).

**Table S2.** Calculation of the sensitivity correction factors for MCP, DCP, and TCP.				
**Compound (m/z)**	**Background (A)**	**Signal (A)**	**Background Subtracted Signal (A)**	**Sensitivity Correction Factor**
Phenol (94)	1.2885×10^{-12}	2.1311×10^{-12}	0.8426×10^{-12}	1
2-Chlorophenol (128)	1.1983×10^{-12}	4.2459×10^{-12}	3.0476×10^{-12}	3.62
2,4-Dichlorophenol (162)	1.1506×10^{-12}	6.1699×10^{-12}	5.0193×10^{-12}	5.96
2,4,6-Trichlorophenol (196)	1.1371×10^{-12}	1.7219×10^{-12}	0.4048×10^{-12}	0.480

3. General Procedure for MIMS Experiments

All reactions were performed in triplicate. The reaction mixtures were found to have a pH of 7 and remained unchanged along the course of the reaction.

3.1 General Procedure for the Uncatalyzed Reaction

Tap water (30 mL) was stirred at 40 °C and the background MIMS response was monitored for 5 minutes before adding PhOH (70 µL of a 10.7 mM stock solution, resulting in 25 µM in the reaction mixture). After the signal of PhOH was monitored for 5 minutes, NaOCl (7 µL, 7 eq.) was added and the reaction followed for a further 20 minutes.

3.2 General Procedure for the Iron Porphyrin Catalysed Reactions

Tap water (30 mL) was stirred at 40 °C and the background MIMS response was monitored for 5 minutes before adding PhOH (70 µL of a 10.7 mM stock solution,

resulting in 25 µM in the reaction mixture). After the signal of PhOH was monitored for 5 minutes, an appropriate amount of catalyst was added (see Table S3). After another 5 minutes, NaOCl (7 µL, 7 eq.) was added and the reaction followed for a further 20 minutes.

Table S3. Stock solution concentrations used for the catalysts tested and the appropriate volumes that were added to achieve a particular catalyst loading.

Catalyst	Stock Solution Concentration (mM)	Desired Catalyst Loading (mol%)	Volume Added to Reaction (µL)
$[(TMPyP)FeCl]Cl_4$	0.33	0.5	11
		1	23
		2	45
		3	68
		4	91
$(NH_4)_4[(TPPS)FeCl]$	0.83	0.5	4.5
		1	9
		2	18
		3	27
		4	36
$FeCl_3$	0.82	4	37

3.3 General Procedure for the Reactions Including a Mix of Catalysts

Tap water (30 mL) was stirred at 40 °C and the background MIMS response was monitored for 5 minutes before adding PhOH (70 µL of a 10.7 mM stock solution, resulting in 25 µM in the reaction mixture). After the signal of PhOH was monitored for 4 minutes, an appropriate amount of $[(TMPyP)FeCl]Cl_4$ was added (see Table S4). Two minutes later, an appropriate amount of $[(TPPS)FeCl](NH_4)_4$ was added (see Table S4). After further 4 minutes, NaOCl (7 µL, 7 eq.) was added and the reaction followed for a further 20 minutes.

Table S4. Stock solution concentrations used for the catalysts tested and the appropriate volumes that were added to achieve a particular catalyst loading.

Catalyst	Stock Solution Concentration (mM)	Desired Catalyst Loading (mol%)	Volume Added to Reaction (µL)
$[(TMPyP)FeCl]Cl_4$	0.33	1	23
		1.5	34
$(NH_4)_4[(TPPS)FeCl]$	0.83	1	9
		1.5	14

4. Additional MIMS Data

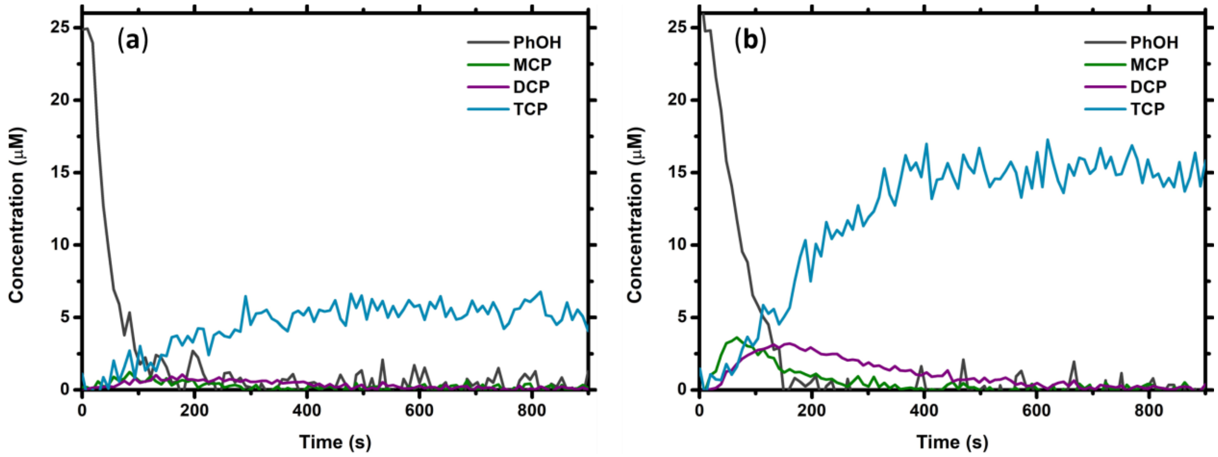

Figure S1. Time trace of the quantified MIMS data showing the depletion of PhOH (dark grey trace, initially 25 μM) after addition of 7 eq. NaOCl at t = 0 s, and the accumulation of MCP, DCP and TCP (green, purple, and blue traces, respectively), in the presence of (**a**) 4 mol% [(TMPyP)FeCl]Cl_4, (**b**) 4 mol% $FeCl_3$. The reaction performed in tap water at 40 °C.

Table S5. Maximum concentrations of MCP, DCP, and TCP upon addition of 7 eq. NaOCl to PhOH (25 μM) in tap water at 40 °C, in the presence of no catalyst, 4 mol% $FeCl_3$, 0.5 – 4 mol% (TMPyP)$FeCl_5$, and 0.5 – 4 mol% (TPPS)FeCl$(NH_4)_4$. Data performed in triplicate and quantified using MIMS.

**Catalyst**	**-**	**$FeCl_3$**	**[(TMPyP)FeCl]Cl_4**					**$(NH_4)_4$[(TPPS)FeCl]**				
**Loading (mol%)**	**-**	**4**	**0.5**	**1**	**2**	**3**	**4**	**0.5**	**1**	**2**	**3**	**4**
**MCP Max. Conc. (μM)**	4.0 ±0.2	3.4 ±0.1	2.3 ±0.4	1.7 ±0.4	1.6 ±0.1	1.2 ±0.1	0.6 ±0.2	2.9 ±0.6	2.3 ±0.6	1.3 ±0.3	0.7 ±0.1	0.5 ±0.1
**DCP Max. Conc. (μM)**	3.3 ±0.1	3.0 ±0.1	2.2 ±0.3	1.8 ±0.2	1.5 ±0.1	1.2 ±0.1	0.7 ±0.2	2.3 ±0.1	2.0 ±0.3	1.0 ±0.3	0.5 ±0	0.4 ±0.1
**TCP Max. Conc. (μM)**	16 ±1	14.9 ±0.3	12 ±1	9 ±2	9 ±1	7.6 ±0.1	4 ±2	12 ±0.4	10 ±2	5 ±2	2.1 ±0.7	1.8 ±0.1

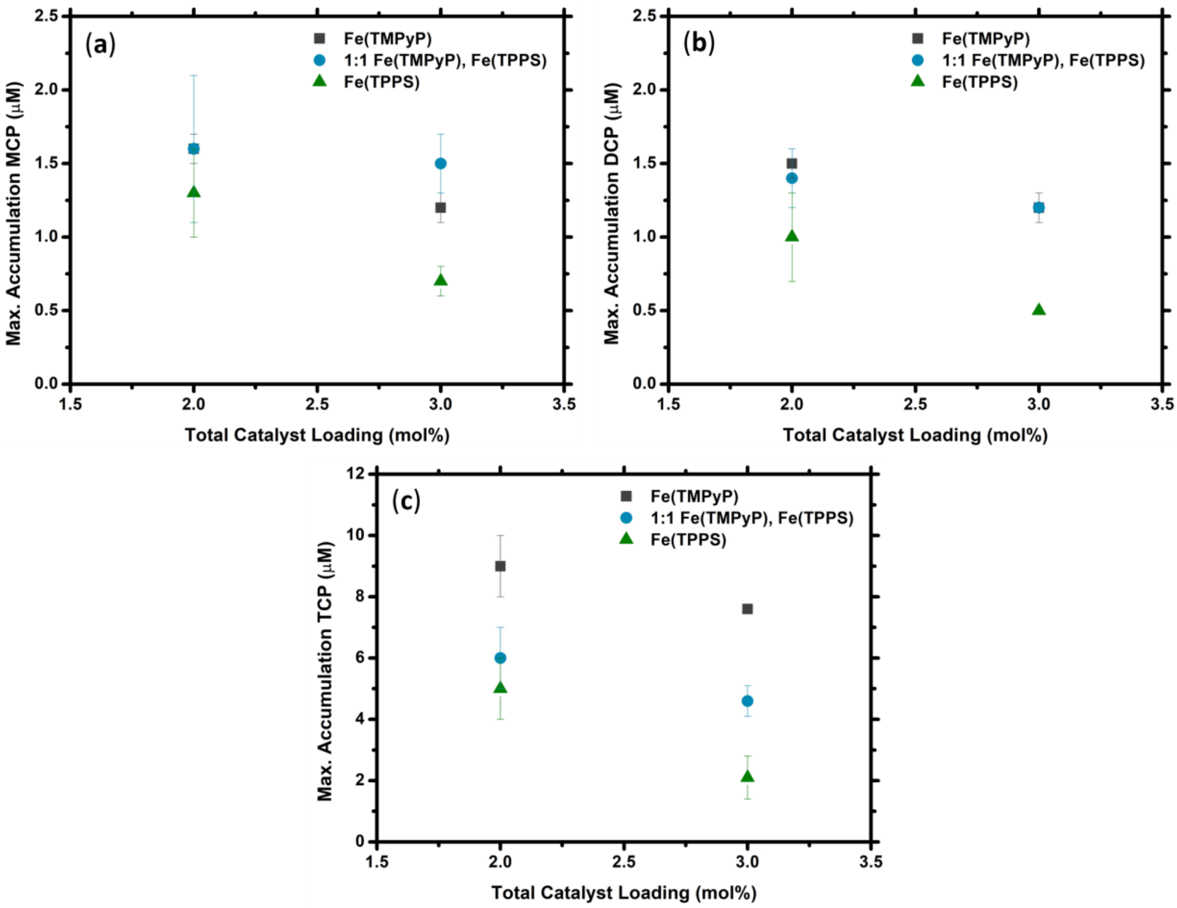

Figure S2. Maximum accumulation of MCP (**a**), DCP (**b**), and TCP (**c**) as a function of total catalyst loading. Data points for $[(TMPyP)FeCl]Cl_4$ are indicated by dark grey squares, those for a 1:1 mix of $[(TMPyP)FeCl]Cl_4$ and $(NH_4)_4[(TPPS)FeCl]$ are indicated by blue circles, the data points for $(NH_4)_4[(TPPS)FeCl]$ are indicated by a green triangle. Reactions were performed in tap water at 40 °C with an initial PhOH concentration of 25 µM. NaOCl (7 eq.) was added to initiate the reaction and its progress was followed by MIMS. All data points were performed in triplicate. Exact values can be found in Table S6.

Table S6. Maximum concentrations of MCP, DCP, and TCP upon addition of 7 eq. NaOCl to PhOH (25 µM) in tap water at 40 °C, in the presence of $[(TMPyP)FeCl]Cl_4$ (abbreviated as TMPyP) and $(NH_4)_4[(TPPS)FeCl]$ (abbreviated as TPPS) compared to a 1:1 mixture of both, equating to the same total iron porphyrin content. Data performed in triplicate and quantified using MIMS.

**Catalyst(s) (Loading in mol%)**	**TMPyP (3)**	**TMPyP(1.5) TPPS (1.5)**	**TPPS (3)**	**TMPyP (2)**	**TMPyP (1) TPPS (1)**	**TPPS (2)**
**MCP Max. Conc. (µM)**	1.2 ± 0.1	1.5 ± 0.2	0.7 ± 0.1	1.6 ± 0.1	1.6 ± 0.5	1.3 ± 0.3
**DCP Max. Conc. (µM)**	1.2 ± 0.1	1.2 ± 0	0.5 ± 0	1.5 ± 0.1	1.4 ± 0.2	1.0 ± 0.3
**TCP Max. Conc. (µM)**	7.6 ± 0.1	4.6 ± 0.5	2.1 ± 0.7	9 ± 1	6 ± 1	5 ± 2

5. General Procedures for UV-Vis and Stopped Flow Experiments

5.1 General Procedure for UV-Vis Experiments

A cuvette equipped with a stir bar and filled with 2 mL tap water was placed in the UV-Vis cryostat at 20 °C. The iron porphyrin was added (see Table S7) and the solution stirred. NaOCl (see Table S7) was then added and the reaction followed by UV-Vis spectroscopy.

Table S7. Volume of stock solutions added to achieve desired reaction concentrations.

Component	Desired Reaction Concentration (μM)	Stock Solution Concentration (mM)	Volume Added to Reaction (μL)
$[(TMPyP)FeCl]Cl_4$	20	1	40
$(NH_4)_4[(TPPS)FeCl]$	20	1	40
NaOCl	40	7.64	10.5
	6685	764	17.5

5.2 General Procedure for Stopped Flow Experiments

Two syringes of the stopped flow were filled with 40 μM iron porphyrin and 80 μM NaOCl. These were left to cool down to 7 °C before initiating the reaction. UV-Vis absorption data points were collected every 1 ms for 200 ms in the case of $[(TMPyP)FeCl]Cl_4$ and every 5 ms for 1 s in the case of $[(TPPS)FeCl](NH_4)_4$.

6. Additional Spectroscopic Data

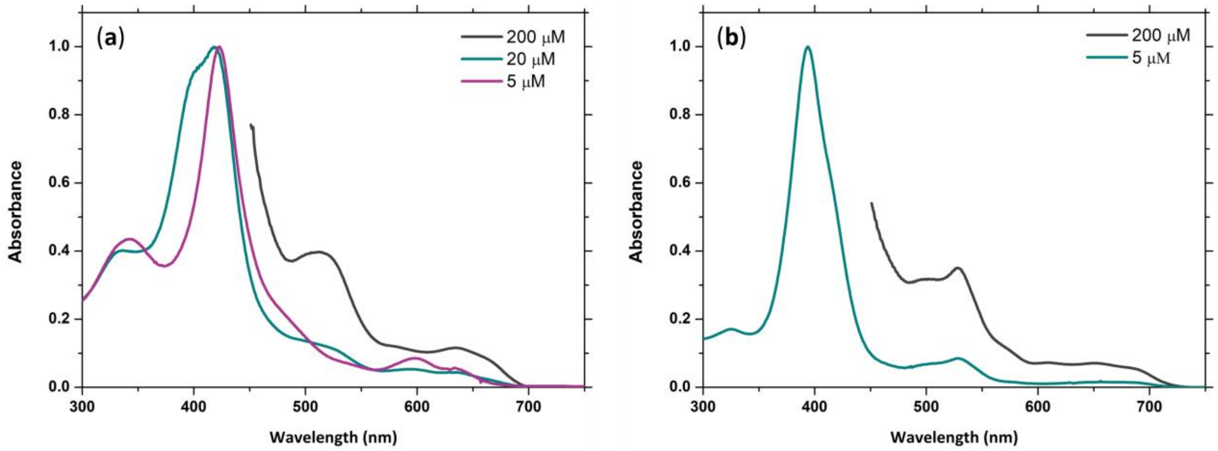

Figure S3. Dilution of (**a**) $[(TMPyP)FeCl]Cl_4$ and (**b**) $(NH_4)_4[(TPPS)FeCl]$ in deionized water. Peak heights of the Soret band (or its saturation in the case of the 200 μM data point) have been normalized for clarity.

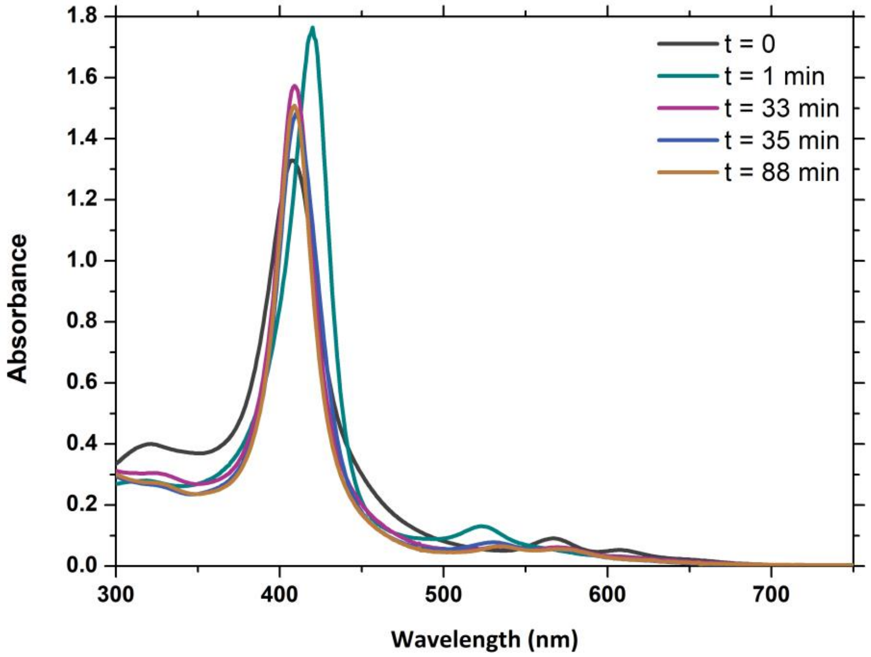

Figure S4. Reaction of $(NH_4)_4$[(TPPS)FeCl] (20 µM) with 2 eq. NaOCl, followed by another 2 eq. NaOCl at t = 34 min, in tap water at 20 °C.

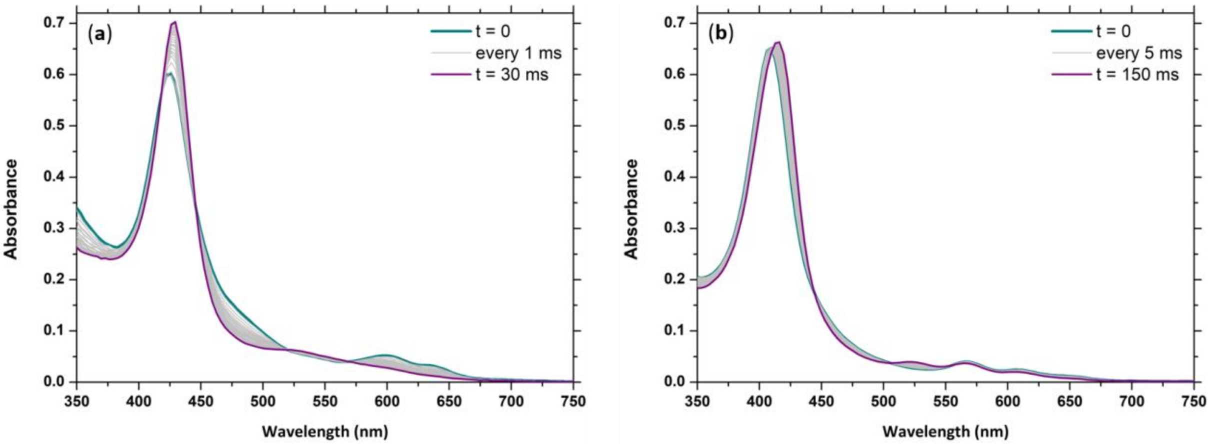

Figure S5. Absorption spectra obtained by stopped flow for the reactions of (**a**) [(TMPyP)FeCl]Cl_4 and (**b**) $(NH_4)_4$[(TPPS)FeCl] with 2 eq. NaOCl at 7 °C in tap water.

7. Data Availability

The raw data associated with this manuscript can be found at https://doi.org/10.34894/IWS45X. The data is organized in different folders by figure number and each folder contains either a scan of the relevant lab journal page or a typed document to explain how to data was collected and labelled. In addition to the presented data, the raw data for some additional experiments is available (see Table S8). The results of these additional experiments are qualitatively similar to those presented in the manuscript.

Table S8. List of additional raw data that can be found in the data archive.

Relevant Folder in Data Archive	Additional Raw data
Figure 4	UV-Vis spectra of the reaction of [(TMPyP)FeCl]Cl_4 (10 µM) with 668 eq. NaOCl in tap water at 20 °C.
	UV-Vis spectra of the reaction of [(TMPyP)FeCl]Cl_4 (20 µM) with 38 eq. NaOCl in tap water at 20 and 5 °C.
	UV-Vis spectra of the reaction of $(NH_4)_4$[(TPPS)FeCl] with 2 eq. NaOCl at 5 °C in tap water.
Figure S3	UV-Vis spectra of [(TMPyP)FeCl]Cl_4 in deionized water in the presence of NaCl.
Figure S5	Stopped flow data for the reaction of [(TMPyP)FeCl]Cl_4 and $(NH_4)_4$[(TPPS)FeCl] with 2 eq. NaOCl in tap water at 20 °C with a total reaction time of 0.2, 0.4, 1, and 4 s. In each case 200 data points are collected within the reaction time.
	Stopped flow data for the reaction of [(TMPyP)FeCl]Cl_4 and $(NH_4)_4$[(TPPS)FeCl] with 2 eq. NaOCl in tap water at 7 °C with a total reaction time of 0.4, 1, and 4 s. In each case 200 data points are collected within the reaction time.

8. References

[1] F. T. Larsen, J. N. McPherson, C. J. McKenzie, F. R. Lauritsen, *Rapid Commun. Mass Spectrom.* **2022**, *36*, e9339.
